# Comparison of Materials in Implant‐Supported Partial Fixed Dental Prostheses: A Randomized Controlled Trial

**DOI:** 10.1111/cid.70129

**Published:** 2026-02-11

**Authors:** Jan Kowar, Alberto Turri, Victoria Stenport, Christina Stervik, Sargon Barkarmo

**Affiliations:** ^1^ Brånemark Clinic, Public Dental Health Care Service Region of Västra Götaland Gothenburg Sweden; ^2^ Department of Prosthetic Dentistry/Dental Material Science Institute of Odontology, The Sahlgrenska Academy at University of Gothenburg Gothenburg Sweden; ^3^ Department of Oral and Maxillofacial Radiology Institute of Odontology, The Sahlgrenska Academy at University of Gothenburg Gothenburg Sweden

**Keywords:** dental implants, dental prosthesis, dental restoration failure, implant‐supported, prosthodontics, randomized controlled trials as topic, titanium, zirconium

## Abstract

**Objectives:**

This randomized controlled trial compared the clinical performances after 1 year of implant‐supported, partial fixed dental prostheses (FDPs) fabricated from monolithic high‐translucency zirconia, veneered high‐translucency zirconia, and titanium‐ceramic.

**Material and Methods:**

Forty‐nine adult participants who required posterior implant‐supported FDPs were randomly assigned to three groups: monolithic zirconia (MZ, *n* = 23 FDPs), veneered zirconia (VZ, *n* = 20 FDPs), and veneered titanium (TC; *n* = 20 FDPs). Clinical and radiographical evaluations were performed at the 1‐year follow‐up, assessing prosthetic and implant survival, biological and technical complications, and marginal bone levels.

**Results:**

A total of 59 FDPs were examined at follow‐up (MZ, *n* = 21; VZ, *n* = 20; TC, *n* = 18). The overall prosthetic survival rate was 96.8%. One FDP in the VZ group failed due to a framework fracture, and one FDP in the TC group was replaced due to extensive chipping. Survival rates were 100% for MZ, 95% for VZ, and 95% for TC. Technical complications occurred in 6.8% of the FDPs, with chipping being the most frequent complication in the TC (11.1%) and VZ (5.0%) groups. Screw loosening occurred in one MZ FDP (4.8%). No chipping was observed in the MZ group. The overall implant survival rate was 99.2%. Biological complications were minimal, with peri‐implant mucositis observed in 8.3% of the FDPs. There were no statistically significant differences in marginal bone level changes between the groups.

**Conclusions:**

Short‐span, implant‐supported, partial FDPs composed of monolithic zirconia, veneered zirconia, and titanium‐ceramic demonstrate high clinical survival rates at the 1‐year follow‐up in posterior regions. Longer‐term studies are needed to confirm the long‐term durability and clinical performance levels of these FDPs.

**Trial Registration:** https://clinicaltrials.gov/study/NCT05296291.

## 1 Introduction

Implant‐supported, partial fixed dental prosthesis (FDP) is frequently being used to rehabilitate partial edentulism. This treatment has improved oral function for many patients who are unwilling or unable to use removable dentures, and it also serves as an excellent alternative when a tooth‐supported bridge is not feasible or appropriate. Treatment with an implant‐supported FDP results in a high survival rate in the long term, and is regarded as a reliable and safe treatment option [1, 2, 3]. However, most studies have focused on implant outcomes, and there are only a few reports on the performance of the prosthetic materials used and the complications related to the prosthesis super‐structure.

Metal‐ceramic restorations supported by implants have long been considered the gold standard for treating partially edentulous patients. Laser‐welded titanium frameworks were introduced more than three decades ago as an alternative to gold alloy casting. Titanium offers advantages such as reduced cost, new fabrication techniques such as replacing traditional casting with CAD/CAM‐milled frameworks, and the use of the same material as the implant [4, 5].

In recent years, zirconia‐based, implant‐supported FDP constructions have been used increasingly as an alternative to metal‐ceramic FDPs [6, 7]. One of the key benefits of zirconia is its superior esthetics compared with metal‐ceramics, as it more accurately mimics the natural coloration of the teeth. In addition, zirconia is biocompatible and exhibits high strength [8, 9]. However, chipping of the veneering porcelain on zirconia‐based restorations is a common cause of failure. Several studies have reported high rates of veneer chipping with zirconia, indicating a frequently observed clinical complication for both tooth‐ and implant‐supported fixed dentures [10, 11, 12]. However, an anatomically guided design of the zirconia framework has been shown to confer significantly higher fracture resistance as compared to a flat design [13].

A rapid development of zirconia materials has led to the introduction of monolithic high‐translucency zirconia, offering enhanced esthetics compared to traditional zirconia [14]. The translucent zirconia allows light to pass through the material naturally, and it can be colored in various shades to mimic the colors of natural teeth. The major advantage of monolithic zirconia (MZ) is that it does not require a veneering porcelain layer, thereby avoiding the commonly seen chipping fractures [15]. In addition, by excluding the veneering porcelain, the material thickness of the zirconia can be increased, resulting in constructions with larger connector dimensions and, consequently, higher fracture strength [16].

Zirconia in dentistry has primarily been tested and evaluated in vitro, and most of the clinical studies to date that have evaluated the outcomes of zirconia have been in relation to tooth‐supported FDPs. As yet, only a few clinical studies on implant‐supported zirconia FDPs have been published, even though it is a commonly used treatment alternative. In addition, there are no controlled clinical studies known to the authors that have compared partial implant FDPs made of zirconia, both veneered and monolithic, with metal‐based FDPs within the same study.

In a review article by Sailer et al., the frequency of survival and complication rate for implant‐supported zirconia‐ceramic FDPs and MZ FDPs was compared with those of metal‐ceramic FDPs [17]. Overall, 16 studies on metal‐ceramics met the inclusion criteria, whereas only 3 studies on zirconia frameworks did so. Despite the low number of articles included in the review, it was concluded that implant‐supported FDPs with veneered zirconia (VZ) could not be recommended as first choice due to the high risks of framework fractures and chipping. Thus, knowledge in this area is restricted due to the limited number of clinical studies that have been carried out on implant‐supported FDPs made of zirconia. Therefore, it is of great importance to conduct controlled clinical studies comparing different prosthetic materials. Such studies can provide information regarding the complications associated with material selection and identify the most‐suitable materials for use in partial implant FDPs, enabling accurate prognostic assessments.

The aim of the present randomized controlled study was to compare the clinical performances of implant‐supported partial fixed dental prostheses (FDPs) fabricated from monolithic high‐translucency zirconia, veneered high‐translucency zirconia, or titanium‐ceramics.

## 2 Materials and Methods

### 2.1 Study Design and Participants

This prospective randomized controlled trial (RCT) was conducted at the Brånemark Clinic in Göteborg (Folktandvården, The region of Västra Götaland), Sweden, from 2020 to 2022. Adults who required permanent, implant‐supported FDPs in the posterior regions of the dentition were considered eligible for inclusion. Thus, all restorations were short‐span, noncantilevered, posterior implant‐supported FDPs.

All patients signed an informed consent form before participation, and the study was conducted in compliance with the Declaration of Helsinki (2013 amendment). All collected data were pseudoanonymized using a code list to ensure patient confidentiality. The trial was approved by the Swedish Ethical Review Authority (Dnr. 2019‐01324) and registered on February 28, 2022 in the clinicaltrials.gov database (ID: NCT05296291). The study start date was September 1, 2020. The recommendations of the CONSORT 2010 Statement for RCTs were followed [18, 19].

**FIGURE 1:**
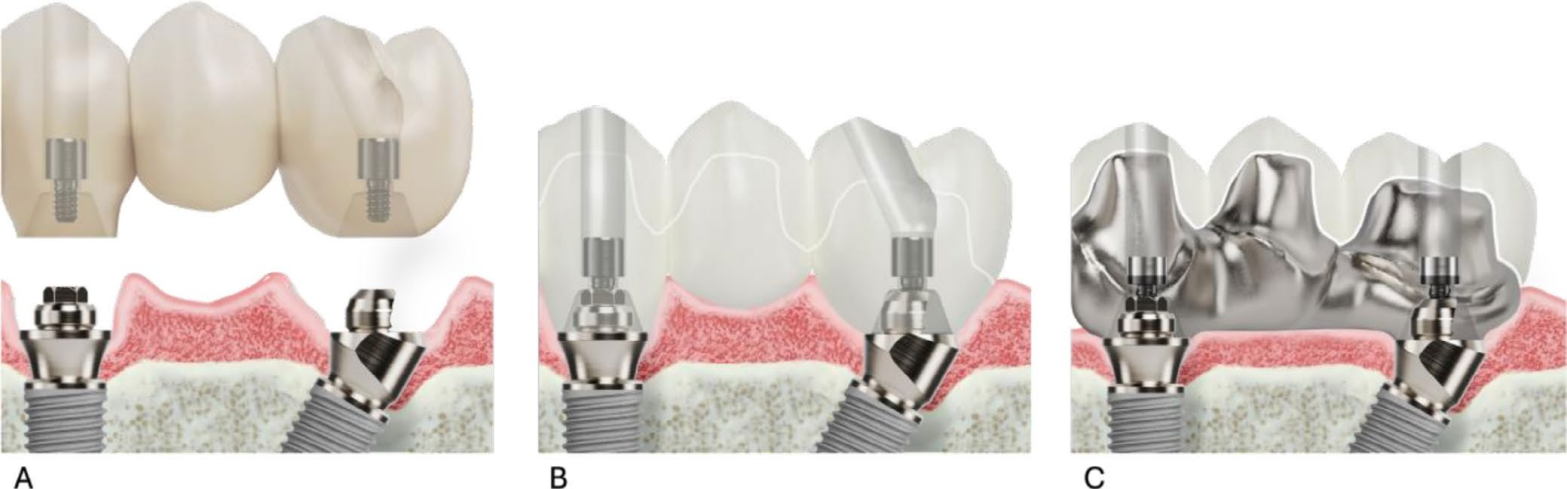
Illustrations of different prosthetic materials: (A) Monolithic zirconia (MZ); (B) veneered zirconia (VZ); and (C) Titanium‐ceramic (TC). *Copyright Nobel Biocare*.

Participants were randomized into three groups using Microsoft Excel, based on the type of prosthetic material used for their implant‐supported FDP (Figure 1).

- Group MZ: High‐translucency, monolithic, full‐contour zirconia.
- Group VZ: Porcelain‐VZ.
- Group TC: Porcelain‐veneered titanium (TC).

### 2.2 Clinical Protocol

All participants were treated by a team of four surgeons and nine prosthodontists with extensive experience of implant‐supported prostheses. The implants used included the Brånemark System MKIII TiUnite and the NobelParallel Conical Connection TiUnite (Nobel Biocare AB, Göteborg, Sweden). Surgical approaches were selected based on the bone quality and on surgeon preference, and included one‐stage or two‐stage protocols, with bone augmentation performed when necessary (i.e., sinus lift surgery) prior to implant placement. Healing or multiunit abutments were placed at the time of the one‐stage or two‐stage surgery depending on the clinical conditions (Table 1).

**TABLE 1:** Baseline characteristics of the patients.

					*n* (%)	*n* assessed
Patients		Group MZ, *N* = 19^a^	Group VZ, *N* = 19^a^	Group TC, *N* = 19^a^	Total, 49 (100%)	
Gender	Female	6^a^ (31.6%)	9^a^ (47.4%)	8^a^ (42.1%)	21 (42.9%)	49
	Male	13^a^ (68.4%)	10^a^ (52.6%)	11^a^ (57.9%)	28 (57.1%)	
Age (years)	Mean ± SD	65.0 ± 14.17	60.1 ± 14.20	58.2 ± 16.88	61.7 ± 15.34	49
	Range	32.2–85	28.1–81.2	20.6–82.3	20.6–85	
General health	ASA I	7 (36.8%)	4 (21.1%)	6 (31.6%)	13 (1.4%)	49
	ASA II	11 (57.9%)	12 (63.2%)	9 (47.4%)	29 (63.0%)	
	ASA III	1 (5.3%)	3 (15.8%)	4 (21.1%)	7 (31.5%)	
	ASA IV	—	—	—	—	
Smoking habit	Smoking	1 (5.3%)	6 (31.6%)	4 (21.1%)	9 (18.4%)	49
	Snuff	2 (10.5%)	2 (10.5%)	—	4 (8.2%)	
	No	16 (84.2%)	11 (57.9%)	15 (78.9%)	36 (73.5%)	
Causes of edentulism	Fracture	1 (4.3%)	1 (5.0%)	2 (10.0%)	4 (6.3%)	63^b^
	Caries	12 (52.2%)	2 (10.0%)	11 (55.0%)	25 (39.7%)	
	Periodontitis	3 (13.0%)	7 (35.0%)	1 (5.0%)	11 (17.5%)	
	NA^c^	7 (30.5%)	10 (50%)	6 (30.0%)	23 (36.5%)	

Prosthetics		Group MZ, *n* = 23	Group VZ, *n* = 20	Group TC, *n* = 20	Total, 63 (100%)	
Number of implants per bridge	2	23 (100%)	20 (100%)	18 (90%)	61 (96.8%)	63
	3	0	0	2 (10%)	2 (3.2%)	
Number of unit bridge	2‐unit	14 (60.9%)	16 (80.0%)	12 (60.0%)	42 (66.7%)	63
	3‐unit	9 (39.1%)	4 (20.0%)	8 (40.0%)	21 (33.3%)	
Surgical protocol	One‐stage	9 (39.1%)	9 (45.0%)	10 (50.0%)	28 (44.4%)	63
	Two‐stage	14 (60.9%)	11 (55.0%)	10 (50.0%)	35 (55.6%)	
Bone augmentation	No	14 (60.9%)	14 (70.0%)	16 (80.0%)	44 (69.8%)	63
	Yes, Sinus lift	8 (34.8%)	5 (25.0%)	2 (10.0%)	15 (23.8%)	
	Yes	1 (4.3%)	1 (5.0%)	2 (10.0%)	4 (6.3%)	
Type of tooth gap	2–3‐tooth gap	8 (34.8%)	8 (40%)	7 (35.0%)	23 (36.5%)	63
	4‐tooth gap	1 (4.3%)	0 (0.0%)	0 (0.0%)	1 (1.6%)	
	Free‐end saddles	14 (60.9%)	12 (60.0%)	13 (65.0%)	39 (61.9%)	
Type of antagonist	Artificial teeth with ceramics	5 (21.7%)	2 (10.0%)	3 (15.0%)	10 (15.9%)	63
	Artificial teeth with ceramics and implants	—	1 (5.0%)	—	1 (1.6%)	
	Implants	6 (26.1%)	4 (20.0%)	2 (10.0%)	12 (19.0%)	
	Natural teeth	9 (39.1%)	8 (40.0%)	9 (45.0%)	26 (41.3%)	
	Natural teeth and implants	2 (8.7%)	3 (15.0%)	5 (25.0%)	10 (15.9%)	
	Removable dentures	1 (4.3%)	2 (10.0%)	1 (5.0%)	4 (6.3%)	

Implants		Group MZ, *n* = 46	Group VZ, *n* = 40	Group TC, *n* = 42	Total, 128 (100%)	
Implant system	Brånemark System Mk III TiUnite	34 (73.9%)	22 (55.0%)	30 (71.4%)	86 (67.2%)	128
	NobelParallel CC TiUnite	12 (26.1%)	18 (45.0%)	12 (28.6%)	42 (32.8%)	
Implant position	Maxilla	30 (65.2%)	26 (65.0%)	20 (47.6%)	76 (59.4%)	128
	Upper right quadrant	16 (34.8%)	16 (40.0%)	9 (21.4%)	41 (32.0%)	
	Upper left quadrant	14 (34.8%)	10 (25.0%)	11 (26.2%)	35 (27.3%)	
	Mandible	16 (34.8%)	14 (35.0%)	22 (52.4%)	52 (40.6%)	
	Lower left quadrant	12 (26.1%)	10 (25.0%)	10 (23.8%)	32 (25.0%)	
	Lower right quadrant	4 (8.7%)	4 (10.0%)	12 (28.6%)	20 (15.6%)	

^a^ Eight subjects with multiple FDPs were included in more than one material group.

^b^ Assessed at the bridge level.

^c^ NA, not available.

All impressions for the FDPs were taken at the abutment level on multiunit abutments using either polyether or silicone impression materials, employing the open‐tray technique with disposable trays. The opposing jaw impression was taken with alginate, and bite registration was performed using either silicone or wax.

The laboratory procedures were conducted at the same dental laboratory for all the FDPs (Tic DP; Nordentic AB, Gothenburg, Sweden). The frameworks were designed by the dental technician using CAD software. The zirconia frameworks were designed with DTX Studio Lab version 1.12.3.1 (Nobel Biocare AB), and the titanium frameworks were designed with the 3Shape Dental System software (3Shape A/S, Copenhagen, Denmark).

Patients in Group MZ received FDPs fabricated from high‐translucency, monolithic, full‐contour zirconia stabilized with 3 mol% yttria. The restorations were produced using the NobelProcera Zirconia Implant Bridge (Nobel Biocare) and manufactured from Nacera Pearl Multi‐Shade zirconia (DOCERAM Medical Ceramics GmbH, Dortmund, Germany). In Group VZ, patients received FDPs with a porcelain‐VZ framework. The zirconia used in this group was the same material as that used for the MZ frameworks in Group MZ. In Group TC, the patients received FDPs with a porcelain‐TC framework. The framework consisted of a NobelProcera Titanium Implant Bridge, Grade 2 (Nobel Biocare).

The designs and dimensions of the frameworks were in accordance with the guidelines set by Nobel Biocare. The connector dimensions of the frameworks depended on the distance between the implants and were the same for all materials. The minimum requirements for connector dimensions were: height of 4.0 mm, width in the range of 2.5–3.0 mm, and cross‐sectional area in the range of 4.95–5.95 mm. All the frameworks were manufactured and milled at the production center of Nobel Biocare in Mahwah, NJ, USA.

The zirconia frameworks in the VZ group were veneered using a conventional full ceramic‐layering technique with the Initial Zr‐FS system (GC Europe N.V., Leuven, Belgium), which completely covered the frameworks. The titanium frameworks were initially coated with a titanium bonding agent (Initial Ti Bonder; GC Europe), followed by veneering with the GC Initial Titanium porcelain system (GC Europe). Each layer was fired separately, in accordance with the manufacturer's instructions. The MZ FDPs were glazed using a glaze paste (IPS Ivocolor Glaze Paste FLUO; Ivoclar Vivadent, Schaan, Liechtenstein).

All the FDPs were screw‐retained on the abutment level with either a straight or an angulated screw channel (ASC). The prosthetic screws were tightened to 15 Ncm, and the screw channels were sealed with silicone or Teflon tape, followed by a layer of composite Tetric EvoCeram (Ivoclar Vivadent) on top.

Participants were followed up at 1 year after final prosthetic delivery (for FDPs), with subsequent evaluations planned at 3 and 5 years. Follow‐up data included clinical and intraoral radiographical evaluations, focusing on prosthetic and implant survival, biological and technical complications, and marginal bone levels (MBLs).

### 2.3 Outcome Measures

The primary outcome was the clinical performance of the experimental groups (MZ and VZ), as compared with the control group (TC). The assessment included prosthetic survival and the incidence rates of biological and technical complications. Survival was adjudged when the restoration remained in place, even in the presence of complications, provided that these did not affect its function.

Failure was defined as the removal of the restoration, either due to implant loss or because the complications were severe enough to require its removal. Technical complications assessed included: framework fractures, chipping, screw loosening, and prosthetic failures.

Secondary outcomes included implant survival, biological complications and changes in MBL. MBLs were measured from periapical radiographs taken at the time of definitive restoration delivery (used as radiographical baseline) and thereafter at the 1‐year clinical examination. MBLs were determined as the distance from the implant platform (reference point) to the most‐apical level of the bone. Measurements were performed mesially and distally, with the average recorded as the MBL. Negative values indicated bone levels below the implant platform, while positive values indicated bone levels above the implant platform. Marginal bone level changes (MBLCs) were calculated as the difference in MBL values between the final prosthetic delivery and the 1‐year follow‐up, as assessed radiographically.

### 2.4 Statistical Analysis

Sample size was determined to detect clinically relevant differences in major technical complications (pronounced ceramic fracture/chipping or framework fracture requiring repair or replacement) between groups at longer follow‐up. Based on published 5‐year complication estimates for implant‐supported FDPs (metal–ceramic ≈11.6% pronounced ceramic fracture/chipping and substantially higher rates reported for zirconia‐ceramic FDPs), we considered a difference of approximately 2% (MZ) versus 40% (VZ) to be clinically meaningful [17]. With 20 FDPs per group, this provides approximately 80% power (two‐sided *α* = 0.05) to detect such a difference. The present paper reports the 1‐year outcomes, while 3‐ and 5‐year follow‐ups are ongoing.

Descriptive statistics were used to summarize the data. Incidence data were tested with Wald tests and chi‐square tests. In addition, multivariate models that included covariates age, number of implants, and implant dimensions were performed.

The statistical analysis was performed in SPSS ver. 29.0.0.0 software (IBM) with standard algorithms.

## 3 Results

### 3.1 Study Population and Baseline Characteristics

**FIGURE 2:**
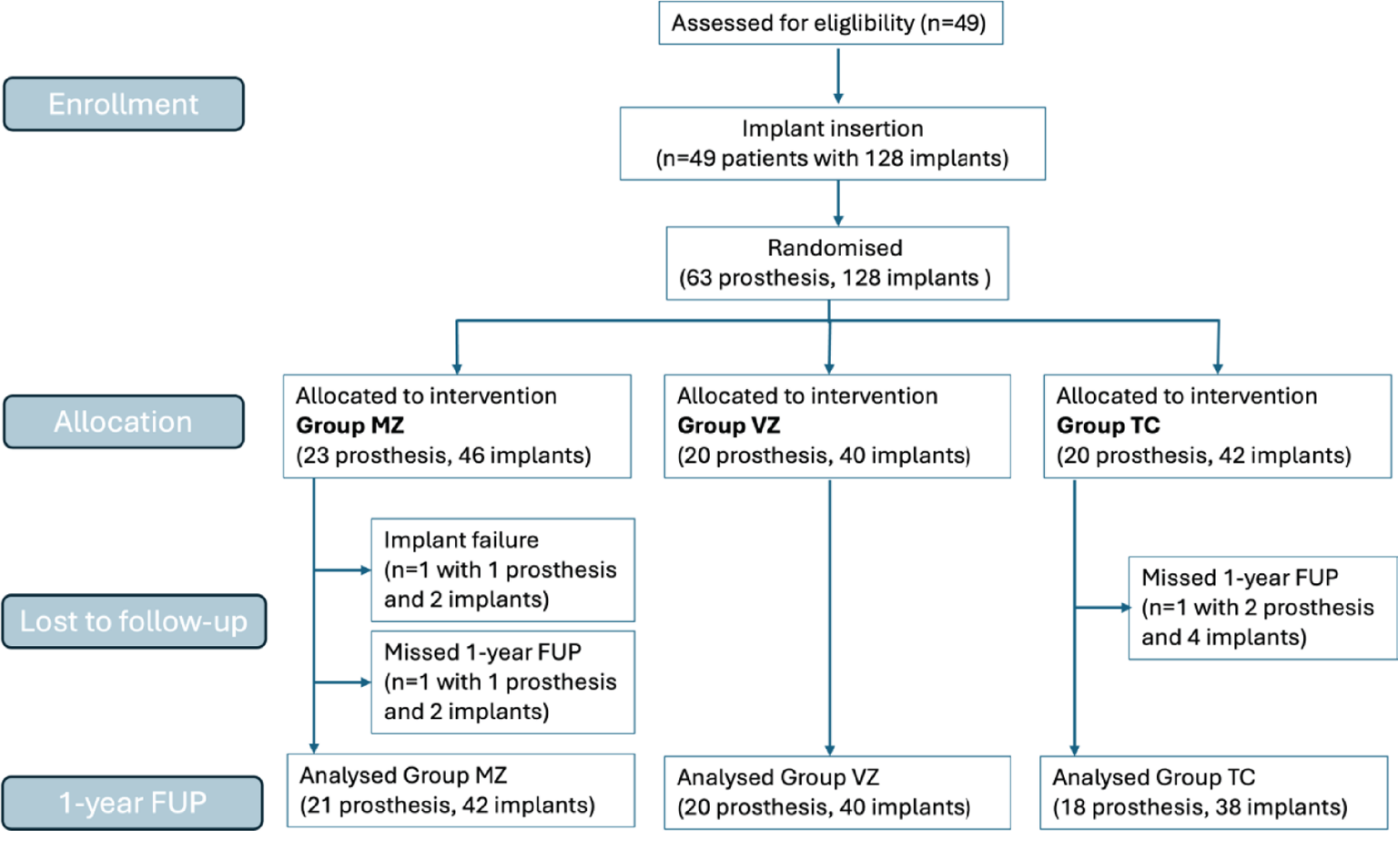
Flow chart of the enrolment process and 1‐year FUP (follow‐up). MZ, monolithic zirconia FDP; VZ, veneered zirconia FDP; TC, titanium‐ceramic FDP; FUP, follow‐up.

A total of 49 participants (28 males and 21 females) were included in the study, with a mean age of 61.7 ± 15.3 years (range 20.6–85 years). Participants were randomized to receive one of three FDP types: Group MZ, 23 FDPs; Group VZ, 20 FDPs; and Group TC, 20 FDPs (Figure 2).

All subjects received the final prosthesis. Of the 49 subjects, 36 (73.5%) received one prosthesis, 12 subjects (24.5%) received two prostheses, and one (2.0%) subject received three prostheses. Subjects with multiple prostheses may have been included in more than one material group (Table 2).

**TABLE 2:** Number of FDPs per subject.

Subjects with	*N*	%
One prosthesis	36	73.5
Two prostheses	12^a^	24.5
Three prostheses	1^a^	2.0
Total	49	100.0

^a^ Eight subjects with multiple FDPs were included in more than one material group.

The final prosthetic delivery occurred at a mean (±SD) of 4.82 ± 1.89 months after implant insertion. In total, 63 FDPs were delivered to the patients, with the majority (*n* = 42, 66.7%) consisting of 2‐unit FDPs, and the remainder (*n* = 21, 33.3%) being 3‐unit FDPs. Most of the rehabilitated regions had no teeth present posterior to the FDP (*n* = 39, 61.9%) and were classified as “free‐end saddles.”

The type of final restoration antagonist was also assessed, and the complete antagonist status distribution is shown in Table 1.

The surgical protocols included both one‐stage (*n* = 28, 44.4%) and two‐stage (*n* = 35, 55.6%) approaches, with bone augmentation performed in 19 (30.2%) cases. Bone augmentation procedures consisted of sinus lift surgery (*n* = 15, 23.8%), which was chosen when it was judged that optimal clinical stability of the implant could not be achieved immediately. All implants were placed in the posterior region, except for one that was placed at position 23 (0.8%) in a subject in Group MZ. Most of the implants were inserted in the maxilla (*n* = 76, 59.4%), while 52 (40.6%) implants were placed in the mandible. The analysis per prosthetic group demonstrated that while for Groups MZ and VZ, most implants were indeed inserted in the maxilla (*n* = 30, 65.2% and *n* = 26, 65.0%, respectively), in Group TC the implants were more frequently placed in the mandible (*n* = 22, 52.4%).

### 3.2 Clinical and Radiographical Follow‐Up

The 1‐year follow‐up examinations were performed by prosthodontists or dental hygienists, following a standardized clinical and radiographic evaluation protocol. Forty‐six participants and 59 FDPs were evaluated at the 1‐year follow‐up, which was conducted at a mean of 13.3 ± 3.1 months (range 4.8–30.4 months) after final prosthetic delivery. Radiographical evaluation included 117 implants with high‐quality radiographs available for bone‐level analysis.

### 3.3 Prosthetic Survival

At the 1‐year follow‐up, the overall prosthetic survival rate was 96.8%. In Group TC, the survival rate was 95%. The FDP in this group exhibited a large chipping fracture that exposed the metal and was esthetically undesirable, necessitating the replacement of the entire prosthesis with an all‐ceramic FDP.

Groups MZ and VZ demonstrated prosthetic survival rates of 100% and 95.0%, respectively. One prosthesis in Group VZ failed due to a framework fracture.

### 3.4 Biological Complications

**FIGURE 3:**
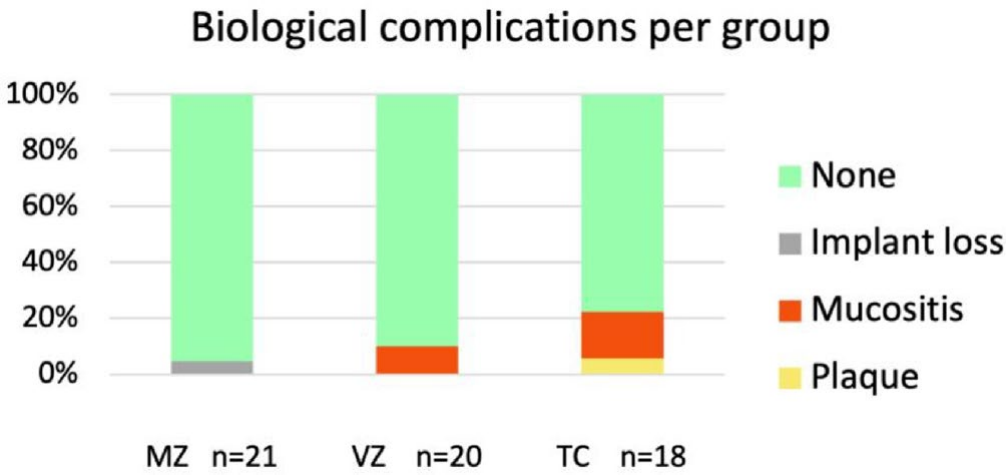
Biological complications (%) by group at the 1‐year follow‐up. MZ, monolithic zirconia; VZ, veneered zirconia; TC: titanium ceramic. No statistically significant differences were observed between the groups (*p* > 0.05).

Biological complications were minimal (Figure 3). In Group MZ, one implant was lost early before loading with an FDP. Peri‐implant mucositis was observed in five cases of FDP (8.3%), which included three (16.7%) in Group TC and two (10.0%) in Group VZ (none in Group MZ). Plaque accumulation was observed in one FDP in Group TC and none in the experimental groups.

### 3.5 Technical Complications

**FIGURE 4:**
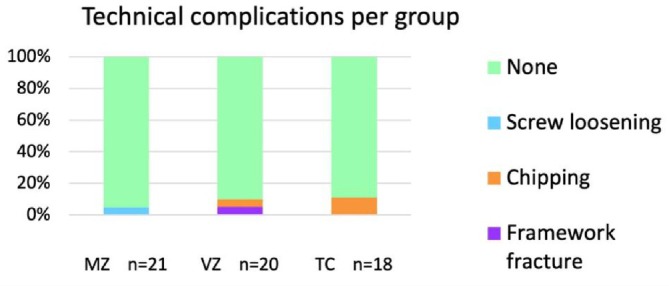
Technical complications (%) by group at the 1‐year follow‐up. MZ: monolithic zirconia; VZ: veneered zirconia; TC: titanium ceramic. No statistically significant differences were observed between the groups (*p* > 0.05).

**FIGURE 5:**
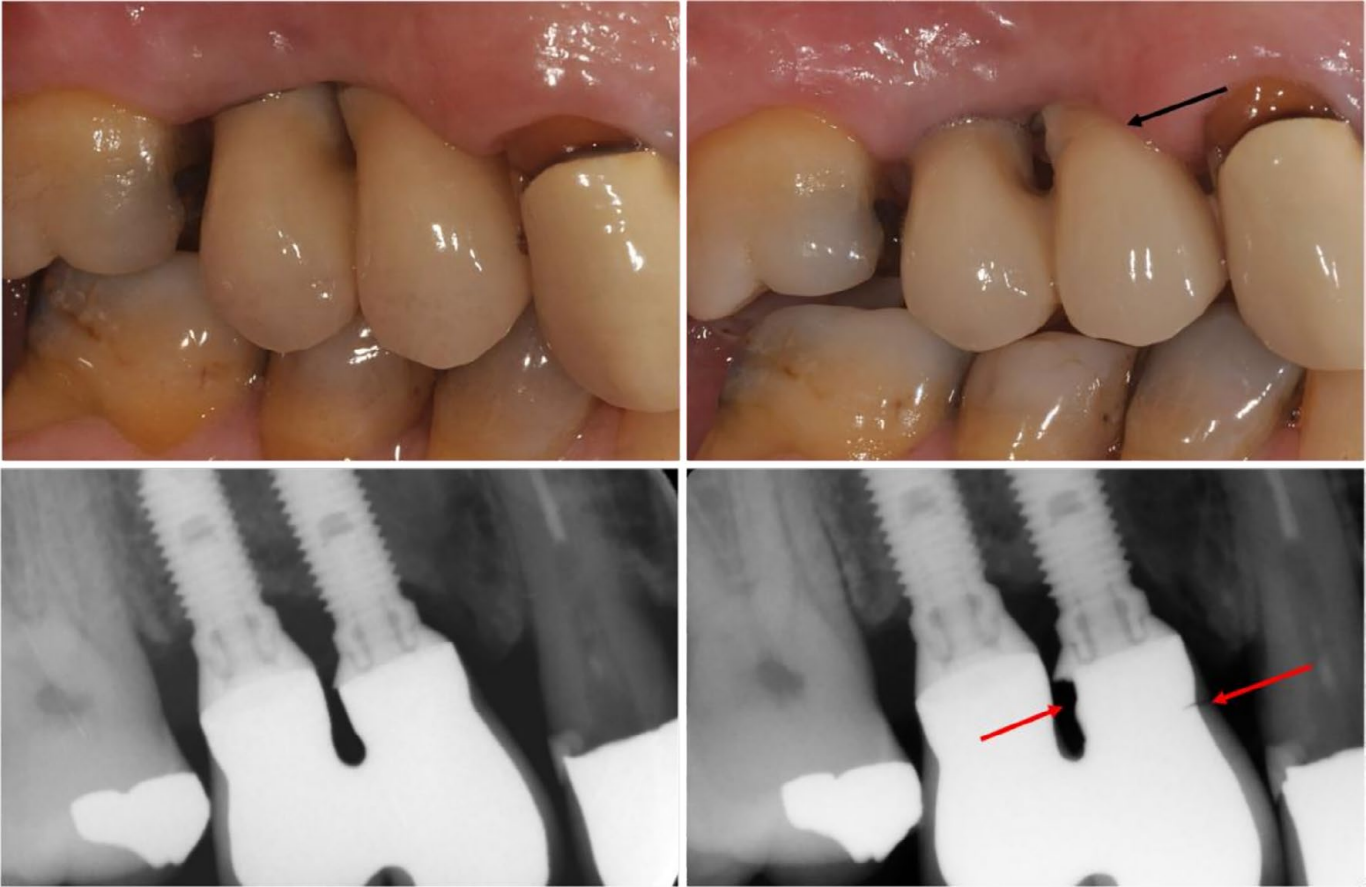
Clinical and radiographical images of a veneered zirconia (VZ) FDP at delivery (left). The upper images show the clinical view, while the lower images present the radiographs. The close proximity of the implants required proximal framework reduction for cleaning access. At the 1‐year follow‐up (right), a framework fracture is visible (arrows).

Technical complications were reported for 6.8% of the FDPs (Figure 4). In Group TC, chipping occurred in two FDPs (11.1%), and no other technical complications were reported. In Group VZ, chipping occurred in one FDP (5.0%), and there was one framework fracture. The fracture in Group VZ extended horizontally into the framework of the FDP, just above the abutment of the mesial implant (Figure 5). In Group MZ, there were no cases of framework fractures or chipping, although screw loosening occurred in one FDP (4.8%).

### 3.6 Implant Survival and MBLCs

The overall implant survival rate at the 1‐year follow‐up was 99.2%, with survival rates of 97.7% in Group MZ and 100% in Groups VZ and TC.

**FIGURE 6:**
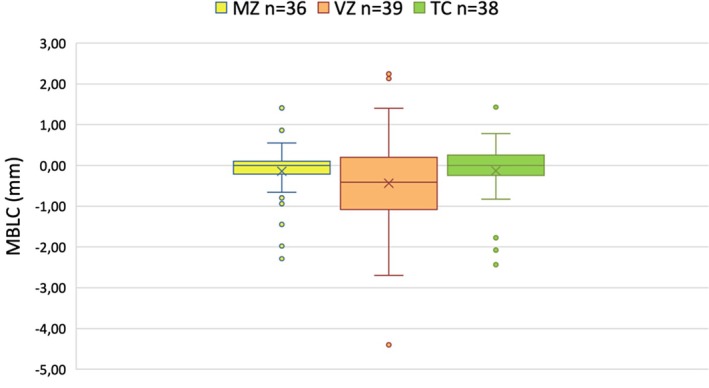
Marginal bone level changes by group at the implant level from the final prosthesis delivery to 1‐year follow‐up. MZ, monolithic zirconia; VZ, veneered zirconia; TC, titanium ceramic.

The mean MBLC for all the material groups was −0.26 ± 0.96 mm. In the Group TC, the mean MBLC was −0.15 ± 0.77 mm. Experimental Groups MZ and VZ demonstrated mean MBLC values of −0.18 ± 0.77 mm and −0.45 ± 1.24 mm, respectively (Figure 6). Although the control group showed slightly less bone loss compared to the experimental groups, these differences were not statistically significant (*p* > 0.05).

## 4 Discussion

This randomized controlled study compared the clinical performances of short‐span, noncantilevered, posterior FDPs made from monolithic or veneered high‐translucency zirconia, as well as titanium‐ceramic. The findings of this study demonstrate that the FDPs made from all three investigated prosthetic materials (MZ, VZ, and TC) exhibit high clinical survival rates during a 1‐year follow‐up period.

The overall prosthetic survival rate was 96.8%, with one prosthesis in Group VZ showing failure due to a framework fracture. In addition, one prosthesis in Group TC was replaced due to an extensive chipping fracture. Despite the short follow‐up period, the results (> 95%) are consistent with those of previous studies reporting high survival rates for both zirconia‐based and metal‐based implant‐supported FDPs [17].

The technical complications in this study affected 6.8% of the FDPs. Chipping was the most frequently observed issue, occurring in 11.1% of the titanium‐ceramic prostheses (Group TC) and 5.0% of the VZ prostheses (Group VZ). High rates of chipping have been reported for TC, tooth‐supported restorations [20]. To avoid porcelain fractures on titanium frameworks, alternative metals such as cobalt‐chromium (CoCr) are used due to their greater stiffness and strong porcelain bonding [21]. However, another clinical study has shown no significant differences in the complications linked to CoCr porcelain FDPs compared to titanium porcelain FDPs [22].

The occurrence of chipping in the VZ FDP in this study aligns with previous findings, emphasizing the vulnerability of veneering porcelain in zirconia‐based restorations [10, 11, 12]. Importantly, no chipping was observed in the MZ group, reinforcing the view that omitting the veneering layer reduces the risk of fracture‐related complications [23, 24].

In this study, the FDP that had a skeletal fracture in Group VZ had implant placements that were positioned very close to each other (Figure 5). To allow space for adequate proximal cleaning, the skeletal framework was reduced in dimension, which may have contributed to the fracture. This suggests that avoiding excessively close implant placement is important for preventing such complications. In addition, if framework reduction is required, a metal framework may be preferable to a ceramic one due to its superior mechanical properties. In a systematic review, tooth‐supported, metal‐ceramic prostheses demonstrated higher survival rates than all‐ceramic FDPs [25].

Another possible approach to prevent such fractures is the use of separate, single implant‐supported crowns, rather than splinted crowns, in cases where the number of implants corresponds to the number of restored units. However, in the present study, all the FDPs were either splinted or with pontic units. Nonsplinted implant restorations offer potential benefits, such as improved access for adequate oral hygiene and enhanced esthetic outcomes. Conversely, splinting implant‐supported FDPs is thought to improve occlusal load distribution, which may reduce mechanical complications, particularly in posterior load‐bearing regions. However, most of the studies advocating splinting have been theoretical in nature and have not clearly demonstrated superior long‐term outcomes compared to single implant crowns [26]. Furthermore, a systematic review has shown that the use of FPDs with or without pontics does not significantly affect the clinical outcomes [27].

Factors such as implant positioning, prosthetic design, and material selection play crucial roles in ensuring the long‐term success and survival of FDPs [3]. In this study, the majority of the FDPs were relatively short‐span, with 67% consisting of two‐unit FPDs and 33% made up of three‐unit FDPs. It has been shown that greater FDP length in both tooth‐ and implant‐supported FDPs carries a higher risk of fracture, as compared with shorter FDPs [22, 28]. On the other hand, partial FDPs lack the cross‐arch stabilization seen in completely edentulous arches, making them more susceptible to bending forces [29]. Although there was a small imbalance in the distribution of two‐ and three‐unit FDPs between the groups, it is unlikely that this has substantially influenced the overall results, given the short‐span nature of the reconstructions and the low incidence of complications. In addition, the FDPs included in this study were located in the posterior regions of the dentition, where occlusal forces are generally higher than in the anterior dentition, thereby subjecting them to a more challenging biomechanical environment [30]. Despite these increased functional demands, the incidence rate of complications remained low.

Another methodological consideration is that several surgeons and prosthodontists from the clinic were involved in the treatments. However, all clinicians adhered to a standardized treatment protocol to promote consistency and minimize interoperator variability.

The findings of this study indicate a low incidence of biological complications with a high overall implant survival rate (99.2%) at the 1‐year follow‐up, which is consistent with the findings of previous studies [1, 2, 3]. Although this study demonstrates low prevalence rates of mucositis over a short period, other studies have reported higher prevalence rates [31]. The MBLCs were comparable across the groups, with no statistically significant differences.

## 5 Conclusion

The findings of this study suggest that partial, implant‐supported FDPs represent a feasible treatment option for tooth loss in the posterior dental regions. Within the limitations of the 1‐year follow‐up, monolithic high‐translucency zirconia, veneered high‐translucency zirconia, and titanium‐ceramic FDPs all demonstrated high survival rates and low incidences of biological and technical complications. However, further long‐term studies are needed to confirm the durability and clinical performance of such FDPs.

## Author Contributions

Jan Kowar, Alberto Turri, Victoria Stenport, and Sargon Barkarmo conceived and designed the study. Jan Kowar, Alberto Turri, Victoria Stenport, and Sargon Barkarmo conducted the clinical investigations. Jan Kowar and Sargon Barkarmo curated and managed the data. Jan Kowar, Christina Stervik, and Sargon Barkarmo performed the data analysis. Jan Kowar, Alberto Turri, Victoria Stenport, Christina Stervik, and Sargon Barkarmo contributed to the methodology and validation of results. Jan Kowar and Sargon Barkarmo developed the software tools and visualizations. Jan Kowar, Victoria Stenport, and Sargon Barkarmo administered the project and acquired the necessary resources and funding. Jan Kowar, Alberto Turri, Victoria Stenport, and Sargon Barkarmo drafted the manuscript. All authors contributed to the review and editing of the final manuscript and approved the submitted version.

## Funding

This work was supported by Nobel Biocare Services AG (Switzerland) (2018‐1563) and Västra Götalandsregionen (TUAGBG‐966246).

## Conflicts of Interest

The authors declare no conflicts of interest.

## Data Availability

The data that support the findings of this study are not publicly available due to privacy or ethical restrictions.
